# A Bayesian Off-Grid DOA Estimation Framework for Close-Angle Scenarios

**DOI:** 10.3390/s26103154

**Published:** 2026-05-16

**Authors:** Wenchao He, Yiran Shi, Hongxi Zhao, Hongliang Zhu, Chunshan Bao

**Affiliations:** 1 School of Mechanical and Electrical Engineering, Changchun Humanities and Sciences College, Changchun 130118, China; hewc23@mails.jlu.edu.cn; 2 College of Communcation Engineering, Jilin University, Changchun 130012, China; hxzhao24@mails.jlu.edu.cn (H.Z.); zhuhl24@mails.jlu.edu.cn (H.Z.); baocs24@mails.jlu.edu.cn (C.B.)

**Keywords:** direction-of-arrival estimation, Bayesian off-grid estimation, sparse Bayesian learning, close-angle source localization, multi-source DOA estimation

## Abstract

Direction-of-arrival (DOA) estimation is a fundamental task in array signal processing and is widely used in radar, sonar, wireless communications, and acoustic localization. Although classical methods such as MUSIC and ESPRIT can achieve high resolution under favorable conditions, their performance often degrades in challenging scenarios involving low signal-to-noise ratios, limited snapshots, and closely spaced sources. To address these difficulties, this paper proposes a Bayesian off-grid DOA estimation framework for close-angle and multi-source scenarios. The proposed method combines multi-measurement-vector evidence learning, diversified candidate construction, and multi-start joint continuous-manifold refinement so that multiple plausible close-angle hypotheses can be preserved and further optimized on the exact angular manifold. In this way, the proposed framework alleviates the source merging caused by high steering-vector coherence and improves estimation robustness in challenging conditions. Experimental results under close-angle, well-separated, varying-snapshot, and three-source settings demonstrate that the proposed method achieves competitive and, in many difficult cases, superior estimation accuracy compared with several representative baseline methods, confirming its effectiveness for robust close-angle DOA estimation.

## 1. Introduction

Direction-of-arrival (DOA) estimation is a fundamental problem in array signal processing and plays an important role in radar, sonar, wireless communications, remote sensing, and acoustic localization. Given multi-sensor observations, the objective is to estimate the incident angles of one or more far-field sources from the received array data. Owing to its broad practical relevance, DOA estimation has been extensively studied for decades, and a large variety of methods have been developed in the literature [1,2]. Among them, classical subspace-based approaches, such as MUSIC [3] and ESPRIT [4], remain widely used because of their clear physical interpretation, relatively mature theoretical foundation, and high-resolution capability under favorable conditions [5,6,7].

Despite their success, conventional DOA estimators often rely on several ideal assumptions. In particular, they usually require sufficiently accurate sample covariance estimation, adequate snapshot support, and moderate source separability. When these conditions are satisfied, subspace decomposition or rotational invariance techniques can provide accurate angle estimates. However, in many practical scenarios, these prerequisites are difficult to guarantee [8,9]. For example, a low signal-to-noise ratio (SNR), limited snapshots, model mismatch, or multiple closely spaced sources can significantly degrade covariance quality and blur the distinction between signal and noise subspaces. As a result, the performance of traditional methods may deteriorate rapidly in challenging environments [10,11,12].

Among the many difficult cases in DOA estimation, close-angle source localization is especially challenging. When multiple sources are located within a narrow angular interval, the corresponding steering vectors become highly coherent, which makes different source components difficult to distinguish. In such cases, conventional peak-search strategies may merge adjacent sources into a single dominant peak, while sequential estimation or deflation-based procedures may accidentally remove energy belonging to neighboring sources [13,14,15]. This problem becomes even more severe in multi-source settings, where the interactions among nearby sources increase the complexity of the estimation landscape. Therefore, improving resolution and robustness in close-angle and high-coherence scenarios remains an important research topic [16,17,18].

Sparse Bayesian learning (SBL) has become an important alternative to classical subspace-based DOA estimation due to its probabilistic sparsity modeling and improved robustness in limited-snapshot regimes. Beyond the standard setting with a perfectly known steering dictionary, several studies have incorporated practical array imperfections into the Bayesian formulation [19,20,21]. A representative example is the work of Dai et al., who considered DOA estimation under unknown mutual coupling and proposed a modified SBL framework based on a hierarchical Student-t prior and EM inference together with SVD-based dimensionality reduction [22]. This line of work demonstrates that Bayesian sparse modeling can be effectively extended to mismatch-calibrated array processing. Nevertheless, such methods are primarily designed to mitigate the manifold of errors induced by mutual coupling, and they do not explicitly focus on the source-merging problem that arises when multiple emitters are closely spaced and the neighboring dictionary atoms become strongly coherent.

Motivated by the above observations, this paper proposes a Bayesian off-grid DOA estimation framework for close-angle and multi-source scenarios. Instead of relying on direct top-peak selection or purely sequential refinement, the proposed method explicitly separates the overall estimation process into three coordinated stages. First, a multi-measurement-vector (MMV)-based Bayesian evidence learning procedure is introduced to exploit the common support structure across multiple snapshots, thereby improving posterior stability under noise and limited data. Second, to prevent closely spaced sources from collapsing into a single dominant hypothesis, an evidence-guided diversified candidate construction strategy is developed to preserve multiple high-quality angle candidates while suppressing redundant selections from the same coherent neighborhood. Third, based on these candidate tuples, a multi-start continuous refinement strategy is performed by directly minimizing a joint residual sum-of-squares objective on the exact array manifold so that multiple source angles can be optimized together rather than separately. In this way, the proposed framework is designed to better handle source coupling, reduce source merging, and improve estimation robustness in high-coherence conditions.

The main contributions of this paper can be summarized as follows. First, we propose a close-angle-oriented Bayesian off-grid estimation framework that explicitly targets the source-merging problem caused by high steering-vector coherence. Instead of treating the MMV Bayesian evidence output as the final decision basis, the proposed framework uses it as an intermediate representation for subsequent candidate preservation and continuous refinement, thereby improving the robustness of close-angle multi-source estimation. Second, we introduce an evidence-guided diversified candidate mechanism tailored to high-coherence close-angle cases. Different from direct top-peak selection, the proposed mechanism preserves several nearby but plausible source hypotheses while reducing redundant selections from the same dominant evidence lobe, thereby alleviating the premature loss of close-angle candidates. Third, we design a multi-start joint continuous-manifold refinement strategy based on a residual sum-of-squares (SSE) objective. Instead of refining source angles independently or sequentially, the proposed strategy jointly optimizes all candidate source angles on the exact array manifold, which improves the stability of multi-source angle estimation and reduces the risk of source merging under strong steering-vector coherence [23]. Extensive experiments under close-angle, well-separated, varying-snapshot, and three-source settings demonstrate the competitive and often superior estimation accuracy of the proposed framework for robust close-angle DOA estimation. All simulations and model implementations were conducted in Python (v3.9).

## 2. Method

The proposed method is organized as a three-stage close-angle DOA estimation framework. First, a Bayesian evidence-learning stage is used to obtain a coarse evidence profile over the angular grid from the MMV observation model. This stage provides a probabilistic indication of plausible source regions but is not directly used for final top-*K* peak selection. Second, an evidence-guided diversified candidate construction stage is introduced to retain multiple plausible close-angle hypotheses and reduce redundant selections from the same dominant evidence lobe. Third, a multi-start joint continuous-manifold refinement stage is performed, where candidate angle tuples are jointly optimized on the exact array manifold by minimizing a residual SSE objective. The overall workflow is therefore “evidence learning–candidate preservation–joint refinement”, which is designed to reduce source merging in high-coherence close-angle scenarios.

### 2.1. Signal Model

Consider a uniform linear array (ULA) with *M* sensors located at normalized positions

(1) $$m={\left[0,1,\ldots ,M-1\right]}^{⊤}\in R^{M}$$

where the inter-element spacing is *d* and the wavelength is λ. Let θ∈R denote an azimuth angle. The steering vector is

(2) $$a\left(\theta \right)=\exp\;\left(-j\;2\pi \frac{d}{\lambda }\;m\;\sin\;\left(\theta \right)\right)$$

where the exponential is taken elementwise.

Across *T* snapshots, the measurement matrix

(3) $$X=\left[x_{1},x_{2},\ldots ,x_{T}\right]\in C^{M\times T}$$

obeys the narrowband far-field model

(4) $$X=A\left(\theta \right)\;S+N,\;\theta ={\left[\theta^{\left(1\right)},\ldots ,\theta^{\left(K\right)}\right]}^{⊤}\in R^{K}$$

Here, $A\left(\theta \right)=\left[a\left(\theta^{\left(1\right)}\right),\ldots ,a\left(\theta^{\left(K\right)}\right)\right]\in C^{M\times K}$, $S\in C^{K\times T}$ collects source snapshots, and $N\in C^{M\times T}$ is noise.

Next, we assume circular complex Gaussian noise with independent and identically distributed columns:

(5) $$n_{t}∼\mathrm{CN}\left(0,\sigma^{2}I_{M}\right),\;t=1,\ldots ,T$$

where $\sigma^{2}>0$ is the noise variance.

Let $\phi ={\left[\phi_{1},\ldots ,\phi_{G}\right]}^{⊤}$ be a coarse angular grid with *G* grid points. Define the grid dictionary

(6) $$A_{g}=\left[a\left(\phi_{1}\right),\ldots ,a\left(\phi_{G}\right)\right]\in C^{M\times G}$$

We normalize each steering vector for numerical stability:

(7) $${\tilde{a}}_{g}=\frac{a(\phi_{g})}{∥a\left(\phi_{g}\right){∥}_{2}},\;\tilde{A}=\left[{\tilde{a}}_{1},\ldots ,{\tilde{a}}_{G}\right]\in C^{M\times G}$$

For a single snapshot, one may write

(8) $$x_{t}=\tilde{A}w_{t}+n_{t},\;w_{t}\in C^{G}$$

However, DOA support is common across snapshots while amplitudes vary. To exploit this structure and stabilize evidence under noise and coherence, we stack all snapshots and obtain the MMV model:

(9) $$X=\tilde{A}\;W+N,\;W=\left[w_{1},\ldots ,w_{T}\right]\in C^{G\times T}$$

The row-sparsity of W captures a small number of active grid angles shared across *T* snapshots.

### 2.2. Bayesian Evidence Learning for Coarse Angular Support

We associate each grid coefficient with a non-negative variance hyperparameter to promote sparsity:

(10) $$\gamma ={\left[\gamma_{1},\ldots ,\gamma_{G}\right]}^{⊤}\in R_{+}^{G},\;\Gamma =\mathrm{diag}\left(\gamma \right)\in R_{+}^{G\times G}$$

Conditioned on γ, MMV columns are independent Gaussian:

(11) $$p\left(W|\gamma \right)=\prod_{t=1}^{T}\mathrm{CN}\left(w_{t};0,\Gamma \right)$$

Under (5), the likelihood factorizes as

(12) $$p\left(X|W,\sigma^{2}\right)=\prod_{t=1}^{T}\mathrm{CN}\left(x_{t};\tilde{A}w_{t},\sigma^{2}I_{M}\right)$$

Then, the posterior is Gaussian:

(13) $$p\left(W|X,\gamma ,\sigma^{2}\right)=\prod_{t=1}^{T}\mathrm{CN}\left(w_{t};\mu_{t},\Sigma \right)$$

with

(14) $$\begin{array}{cc} \mu_{t} & =\Gamma \;{\tilde{A}}^{H}\;C^{-1}x_{t} \end{array}$$

(15) $$\begin{array}{cc} \Sigma & =\Gamma -\Gamma \;{\tilde{A}}^{H}\;C^{-1}\tilde{A}\;\Gamma \end{array}$$

where the evidence covariance C is

(16) $$C=\tilde{A}\;\Gamma \;{\tilde{A}}^{H}+\sigma^{2}I_{M}\in C^{M\times M}$$

For Type-II evidence learning of γ, it is convenient to aggregate the posterior means across snapshots. Define

(17) $$\mu =\left[\mu_{1},\ldots ,\mu_{T}\right]\in C^{G\times T}$$

Integrating out W under the Gaussian prior then yields the marginal likelihood

(18) $$p\left(X|\gamma ,\sigma^{2}\right)=\prod_{t=1}^{T}\mathrm{CN}\left(x_{t};0,C\right)$$

and the log-evidence is

(19) $$L\left(\gamma ,\sigma^{2}\right)=-T\log\;\det\left(C\right)-\sum_{t=1}^{T}x_{t}^{H}C^{-1}x_{t}$$

A standard MMV-SBL update for $\gamma_{g}$ is

(20) $${\hat{\gamma }}_{g}=\frac{1}{T}{∥\mu_{g:}∥}_{2}^{2}+{\left[\Sigma \right]}_{gg},\;g=1,\ldots ,G$$

where $\mu_{g:}\in C^{1\times T}$ is the *g*th row. For stability in weak evidence or high coherence, we use an under-relaxed update:

(21) $$\gamma_{g}\leftarrow \max\left\{\gamma_{\min},\;\left(1-\eta_{\gamma }\right)\gamma_{g}+\eta_{\gamma }{\hat{\gamma }}_{g}\right\}$$

In the close-angle regime, adjacent grid atoms become highly coherent and the ARD evidence may collapse into a single dominant lobe. To counter this effect at the evidence-learning stage, we introduce a local-band coherence matrix built from the normalized dictionary $\tilde{A}$:

(22) $$L_{ij}=\left\{\begin{array}{cc} |{\tilde{a}}_{i}^{H}{\tilde{a}}_{j}{|}^{p}, & |i-j|\leq b,\;i\neq j,\\ 0, & \mathrm{otherwise}, \end{array}\right.\;p\geq 1$$

where *b* is a small bandwidth so that only local high-coherence interactions are penalized. For a ULA, the inner product between two steering vectors depends smoothly on the angular difference. Let $\Delta \theta =\theta -\theta^{\prime }$. Ignoring the degree-to-radian factor for brevity, evaluating this sum leads to the Dirichlet-kernel expression below, showing that the coherence decreases as |Δθ| increases:

(23) $$\frac{{|a\left(\theta \right)}^{H}a\left(\theta^{\prime }\right)|}{{∥a\left(\theta \right)∥}_{2}\;{∥a\left(\theta^{\prime }\right)∥}_{2}}=\frac{1}{M}\left|\sum_{m=0}^{M-1}\exp\;\left(j2\pi \frac{d}{\lambda }m\left(\sin\theta -\sin\theta^{\prime }\right)\right)\right|\approx \frac{1}{M}\left|\frac{\sin\;\left(\frac{M}{2}\Delta \psi \right)}{\sin\;\left(\frac{1}{2}\Delta \psi \right)}\right|$$

where $\Delta \psi =2\pi \frac{d}{\lambda }\left(\sin\theta -\sin\theta^{\prime }\right)$. Therefore, only nearby angles produce strong coherence, while far-away grid atoms have negligible interaction. This motivates the band-limited construction in (22), which focuses the repulsion on close-angle ambiguities and avoids distorting the global evidence landscape. After obtaining the standard update ${\hat{\gamma }}_{g}$ in (20), we apply a normalized repulsive shrinkage:

(24) $$\hat{\gamma }\leftarrow \hat{\gamma }⊘\left(1+\lambda_{\mathrm{rep}}\;\frac{L\hat{\gamma }}{∥L\hat{\gamma }{∥}_{\infty }+\epsilon }\right),\;\lambda_{\mathrm{rep}}\geq 0$$

where ⊘ denotes elementwise division and ϵ>0 is a tiny constant. This operation preserves the global peak location while suppressing redundant activations within a coherent neighborhood, thereby improving the separability of close-angle hypotheses before candidate construction.

Next, we define a non-negative evidence proxy

(25) $$o_{g}=\sqrt{∥\mu_{g:}{∥}_{2}^{2}+T{\left[\Sigma \right]}_{gg}},\;g=1,\ldots ,G$$

which forms peaks around plausible DOAs.

However, when two or more sources are closely spaced, the corresponding steering vectors become highly coherent:

(26) $$\kappa \left(\theta^{\left(i\right)},\theta^{\left(j\right)}\right)=\frac{|a{\left(\theta^{\left(i\right)}\right)}^{H}a\left(\theta^{\left(j\right)}\right)|}{∥a\left(\theta^{\left(i\right)}\right){∥}_{2}\;{∥a\left(\theta^{\left(j\right)}\right)∥}_{2}}\approx 1$$

In this regime, the evidence profile $\left\{o_{g}\right\}$ may exhibit a single dominant mode with a shoulder, and selecting the top-*K* bins can repeatedly sample the same mode. Moreover, sequential deflation may remove energy belonging to other coherent sources due to mismatch. This motivates (i) a diversified candidate set that preserves multiple nearby hypotheses and (ii) joint multi-angle refinement rather than one-by-one updates.

### 2.3. Evidence-Guided Diversified Candidate Construction

To explicitly retain multiple close-angle hypotheses while avoiding redundant sampling from a single evidence lobe, we construct a diversified candidate index set by selecting high-evidence grid points under a minimum separation rule. Let $o={\left[o_{1},\ldots ,o_{G}\right]}^{⊤}$ denote the evidence proxy across the grid and sort indices in descending evidence:

(27) $$\zeta =\mathrm{argsort}\left({\left\{-o_{g}\right\}}_{g=1}^{G}\right)$$

where argsort(·) denotes the index-sorting operation that returns the permutation of {1,…,G} corresponding to the descending order of ${\left\{o_{g}\right\}}_{g=1}^{G}$. Equivalently, $\zeta ={\left[\zeta \left(1\right),\ldots ,\zeta \left(G\right)\right]}^{⊤}$ satisfies

(28) $$o_{\zeta (1)}\geq o_{\zeta (2)}\geq \cdots \geq o_{\zeta (G)}.$$

We then build a candidate index set B⊂{1,…,G} with a minimum angular spacing $\delta_{\mathrm{cand}}>0$:

(29) $$B\leftarrow \emptyset ,\;\mathrm{scan}\;\zeta \left(1\right),\zeta \left(2\right),\ldots ;\;\mathrm{add}\;\zeta \left(q\right)\;\mathrm{if}\;|\phi_{\zeta (q)}-\phi_{g^{\prime }}|\geq \delta_{\mathrm{cand}},\;\forall g^{\prime }\in B$$

While (29) enforces a hard angular spacing, extremely close sources may still reside within one broad evidence lobe. To further avoid repeatedly sampling highly coherent neighbors, we optionally perform a coherence-aware selection within a strong-evidence pool. Let Q denote the indices of the top-*Q* evidence values:

(30) $$Q=\left\{\zeta \left(1\right),\ldots ,\zeta \left(Q_{\mathrm{pool}}\right)\right\},\;Q_{\mathrm{pool}}\geq P_{\mathrm{cand}}$$

We then greedily build B by maximizing a quality–diversity surrogate:

(31) $$g^{⋆}=\arg\max_{g\in Q\setminus B}\left(\log\left(o_{g}+\epsilon \right)\;-\;\tau_{\mathrm{div}}\sum_{g^{\prime }\in B}L_{gg^{\prime }}\right)$$

where $L_{gg^{\prime }}$ is from (22) and $\tau_{\mathrm{div}}\geq 0$ controls the diversity strength. Restricting to Q prevents weak spurious peaks from entering the candidate set, while the coherence penalty reduces near-duplicate picks. In practice, (29) serves as a lightweight default, and (31) is activated only for severe close-angle cases.

A pure diversity rule applied over the whole grid may inadvertently admit weak spurious peaks that are diverse but not supported by the evidence, especially when the evidence is relatively flat at a low SNR. Restricting the search space to the top-$Q_{\mathrm{pool}}$ pool Q in (30) enforces a minimum evidence-quality floor before diversification so that the coherence penalty only re-orders strong hypotheses rather than introducing new weak ones. To ensure sufficient alternatives for diversification while keeping the combinatorial cost bounded, we choose

(32) $$Q_{\mathrm{pool}}=\max\left\{c_{Q}\;P_{\mathrm{cand}},\;Q_{\min}\right\},\;c_{Q}\in \left[4,6\right]$$

so that Q covers the main evidence lobe(s) and their shoulders while keeping the pool compact. Moreover, since the coherence matrix L in (22) is band-limited, the diversity penalty in (31) only acts on locally coherent neighbors. This discourages redundant selections within the same main lobe while avoiding any global “push” toward unrelated angular regions. The greedy selection terminates once $\left|B\right|=P_{\mathrm{cand}}$.

Given the resulting candidate set B, we form multi-source initializations by enumerating unordered *K*-tuples:

(33) $$P_{K}=\{\left(i_{1},\ldots ,i_{K}\right):i_{k}\in B,\;i_{1}<\cdots <i_{K},\;|\phi_{i_{p}}-\phi_{i_{q}}\left|\;\geq \delta_{\mathrm{pair}},\;\forall p\neq q\right\}$$

where $\delta_{\mathrm{pair}}\geq 0$ is an optional separation constraint used to suppress near-duplicate hypotheses; to resolve extremely close sources, it can be set to 0 or a very small value. The total number is bounded by

(34) |PK|≤PcandK

which is tractable for moderate *K* under a modest $P_{\mathrm{cand}}$.

### 2.4. Joint K-Angle Residual SSE Objective

For any *K*-angle vector $\vartheta ={\left[\vartheta^{\left(1\right)},\ldots ,\vartheta^{\left(K\right)}\right]}^{⊤}\in R^{K}$, define

(35) $$A\left(\vartheta \right)=\left[a\left(\vartheta^{\left(1\right)}\right),\ldots ,a\left(\vartheta^{\left(K\right)}\right)\right]\in C^{M\times K}$$

Given ϑ, the snapshot-wise least-squares estimate of S in (4) is

(36) $$\hat{S}\left(\vartheta \right)={\left(A{\left(\vartheta \right)}^{H}A\left(\vartheta \right)\right)}^{-1}A{\left(\vartheta \right)}^{H}X\in C^{K\times T}$$

with a tiny diagonal loading if needed for numerical stability. Next, we define the residual:

(37) $$R\left(\vartheta \right)=X-A\left(\vartheta \right)\hat{S}\left(\vartheta \right)$$

And the SSE objective is

(38) $$J\left(\vartheta \right)={∥R\left(\vartheta \right)∥}_{F}^{2}$$

Our final estimate is obtained by the joint optimization

(39) $$\hat{\theta }=\arg\min_{\vartheta \in R^{K}}\;J\left(\vartheta \right)$$

Compared with single-angle scores or deflation, J(ϑ) evaluates a joint fit of all *K* sources and is therefore more stable under high coherence.

### 2.5. Multi-Start Continuous-Manifold Refinement

Given a scalar function f(u), current *u*, and step h>0, evaluate f(u−h),f(u),f(u+h). A quadratic surrogate yields the update

(40) $$u\leftarrow u+\Delta \left(u\right),\;\Delta \left(u\right)=\frac{1}{2}h\;\frac{f(u-h)-f(u+h)}{f(u-h)-2f(u)+f(u+h)}$$

We accept the update only if it reduces *f*; otherwise, we choose the point among {u−h,u,u+h} that gives the smallest *f*.

Next, let $\vartheta ={\left[\vartheta^{\left(1\right)},\ldots ,\vartheta^{\left(K\right)}\right]}^{⊤}$. At each refinement round, we update each coordinate $\vartheta^{\left(k\right)}$ while holding others fixed. For coordinate *k*, define

(41) $$f_{k}\left(u\right)=J\left({\left[\vartheta^{\left(1\right)},\ldots ,\vartheta^{\left(k-1\right)},u,\vartheta^{\left(k+1\right)},\ldots ,\vartheta^{\left(K\right)}\right]}^{⊤}\right)$$

and apply (40) within a bounded span

(42) $$u\in [\vartheta_{0}^{\left(k\right)}-\rho ,\;\vartheta_{0}^{\left(k\right)}+\rho ]$$

A direct evaluation of J(ϑ) in (38) involves the least-squares amplitudes in (36). Equivalently, letting $P_{A}=A\left(\vartheta \right){\left(A{\left(\vartheta \right)}^{H}A\left(\vartheta \right)\right)}^{-1}A{\left(\vartheta \right)}^{H}$ be the projection onto the column space of A(ϑ), we have

(43) $$J\left(\vartheta \right)={∥(I-P_{A})X∥}_{F}^{2}$$

Thus, each parabolic step only requires evaluating J at a few nearby angle settings. Since *K* is small, forming $A^{H}A$ and solving the K×K system is inexpensive, making the refinement substantially faster than dense scanning.

When two sources are closely spaced, the joint SSE J(ϑ) exhibits strong cross-coupling between the two angles; i.e., the local curvature has significant off-diagonal terms. Formally, near a current iterate, a second-order approximation reads

(44) $$J\left(\vartheta +\Delta \right)\approx J\left(\vartheta \right)+\nabla J{\left(\vartheta \right)}^{⊤}\Delta +\frac{1}{2}\Delta^{⊤}H\left(\vartheta \right)\Delta$$

where H is the Hessian. For a close pair (p,ℓ), the entries $H_{p\ell }$ and $H_{\ell p}$ are typically non-negligible, so updating $\vartheta^{\left(p\right)}$ and $\vartheta^{\left(\ell \right)}$ separately may lead to slow zig-zagging or angle swapping. This motivates a pair-coupled block refinement that updates the two angles in a coupled parameterization.

For two highly coherent sources, updating $\vartheta^{\left(k\right)}$ one by one may suffer from strong parameter coupling and angle swapping. To stabilize refinement under close spacing, we optionally refine a detected close pair $\left(\vartheta^{\left(p\right)},\vartheta^{\left(\ell \right)}\right)$ using a center–separation reparameterization:

(45) $$c=\frac{\vartheta^{\left(p\right)}+\vartheta^{\left(\ell \right)}}{2},\;s=\frac{\vartheta^{\left(\ell \right)}-\vartheta^{\left(p\right)}}{2},\;s\geq s_{\min}\geq 0$$

so that $\left(\vartheta^{\left(p\right)},\vartheta^{\left(\ell \right)}\right)=\left(c-s,\;c+s\right)$. Define the pairwise objective by substituting into the joint SSE:

(46) $$J_{p,\ell }\left(c,s\right)=J\;\left({\left[\vartheta^{\left(1\right)},\ldots ,c-s,\ldots ,c+s,\ldots ,\vartheta^{\left(K\right)}\right]}^{⊤}\right)$$

where all non-pair angles are fixed. We then alternate two 1D parabolic updates on *c* and *s* (each using (40)), constraining *c* within a local span and enforcing $s\geq s_{\min}$ to avoid collapse:

(47) $$c\leftarrow \mathrm{ParabolicMin}\left(J_{p,\ell }\left(c,s\right)\right),\;s\leftarrow \mathrm{ParabolicMin}\left(J_{p,\ell }\left(c,s\right)\right),\;s\in \left[s_{\min},s_{\max}\right]$$

This block refinement better matches the close-angle geometry and empirically improves stability compared with separate coordinate updates.

Here, $\vartheta_{0}^{\left(k\right)}$ is the initialization and ρ>0 is the refinement span. We start with $h_{0}>0$ and shrink it geometrically using a factor $\xi_{h}\in \left(0,1\right)$:

(48) $$h\leftarrow \xi_{h}\;h,\;0<\xi_{h}<1$$

This yields a coarse-to-fine coordinate refinement procedure for a given initial *K*-angle vector. Next, we embed this local refinement into a multi-start strategy by generating multiple initializations from the diversified *K*-tuple set $P_{K}$.

Given $P_{K}$ in (33), for each tuple $\left(i_{1},\ldots ,i_{K}\right)\in P_{K}$, we initialize

(49) $$\vartheta^{\left(0\right)}={\left[\phi_{i_{1}},\ldots ,\phi_{i_{K}}\right]}^{⊤}$$

run coordinate refinement to obtain $\vartheta^{\left(\mathrm{ref}\right)}$, and select the best refined solution by the SSE:

(50) $$\hat{\theta }=\arg\min_{\vartheta \in \{\vartheta^{\left(\mathrm{ref}\right)}\}}\;J\left(\vartheta \right)$$

Finally, we sort $\hat{\theta }$ increasingly to report the DOAs. The overall procedure of the proposed method is summarized in Algorithm 1.
**Algorithm 1** Evidence-Guided Multi-Start Joint Refinement for *K* Sources**Require:** Measurements $X\in C^{M\times T}$; grid ϕ; normalized dictionary $\tilde{A}$; noise variance $\sigma^{2}$; SBL iterations *I*; damping/relaxation $\eta_{\gamma }$; candidate size $P_{\mathrm{cand}}$; candidate/pair constraints $\delta_{\mathrm{cand}},\delta_{\mathrm{pair}}$; source number *K*; refinement span ρ; initial step $h_{0}$; shrink factor $\xi_{h}$; inner parabolic iterations ψ; refinement rounds *Q*; local-band coherence matrix L; repulsion weight $\lambda_{\mathrm{rep}}$; diversity weight $\tau_{\mathrm{div}}$; top-$Q_{\mathrm{pool}}$ pool size $Q_{\mathrm{pool}}$; close-pair threshold $\delta_{\mathrm{close}}$; minimum half-separation $s_{\min}$.**Ensure:** Estimated DOAs $\hat{\theta }\in R^{K}$.  1:**(MMV-SBL)** Initialize $\gamma^{\left(0\right)}≻0$.  2:**for** i=1 to *I* **do**  3:      Compute C by (16).  4:      Compute (μ,Σ) by (14)–(15) and (17).  5:      Update ${\hat{\gamma }}_{g}$ by (20) and apply damping (21).  6:      **(Optional)** Apply local-band repulsive shrinkage (24) using L.  7:**end for**  8:Form evidence proxy o by (25).  9:**(Candidates)** Build candidate set B of size $P_{\mathrm{cand}}$:10:      **(Default)** via (29), enforcing spacing $\delta_{\mathrm{cand}}$ if enabled;11:      **(Optional)** Replace by the coherence-aware rule: restrict to top-$Q_{\mathrm{pool}}$ pool (30), then apply greedy selection (31) with $\tau_{\mathrm{div}}$ and L.12:Build *K*-tuples $P_{K}$ by (33), enforcing $\delta_{\mathrm{pair}}$ and $s_{\min}$.13:Initialize best objective $J^{⋆}\leftarrow +\infty$.14:**for all** $\left(i_{1},\ldots ,i_{K}\right)\in P_{K}$**do**15:       Initialize $\vartheta \leftarrow {\left[\phi_{i_{1}},\ldots ,\phi_{i_{K}}\right]}^{⊤}$ and $h\leftarrow h_{0}$.16:       **for** r=1 to *Q* **do**17:             Identify close pairs $\left\{\left(p,\ell \right):|\vartheta^{\left(p\right)}-\vartheta^{\left(\ell \right)}|\leq \delta_{\mathrm{close}}\right\}$.18:             **for** k=1 to *K* **do**19:                   **if** *k* belongs to some close pair (p,ℓ) **then**20:                         Update $\left(\vartheta^{\left(p\right)},\vartheta^{\left(\ell \right)}\right)$ via center–separation block refinement (45)–(47).21:                   **else**22:                         Update $\vartheta^{\left(k\right)}$ by ψ parabolic steps (40) on $f_{k}\left(u\right)$ in (41) within span (42).23:                   **end if**24:             **end for**25:             Shrink the step size $h\leftarrow \xi_{h}\;h$.26:       **end for**27:       Compute $J_{\mathrm{cur}}\leftarrow J\left(\vartheta \right)$ by (38).28:       **if** $J_{\mathrm{cur}}<J^{⋆}$ **then**29:             $J^{⋆}\leftarrow J_{\mathrm{cur}}$, $\hat{\theta }\leftarrow \vartheta$.30:       **if** **end if**31:**end for**32:Sort $\hat{\theta }$ increasingly and return.

## 3. Experiments and Results

For the proposed Bayesian multi-start joint refinement method, the hyperparameters were selected to balance posterior stability, candidate diversity, and local refinement efficiency. In the MMV-SBL stage, the number of evidence-learning iterations was set to I=80. The under-relaxation factor was set to $\eta_{\gamma }=0.4$, so that the hyperparameter updates remained stable under high dictionary coherence and closely spaced sources.

In the candidate-construction stage, the candidate set size was fixed to $P_{\mathrm{cand}}=35$. The minimum angular spacing for candidate selection was set to $\delta_{\mathrm{cand}}=0.4^{\circ }$, which helps avoid repeatedly selecting nearly duplicated grid points from the same dominant evidence lobe while still preserving close-angle hypotheses. For tuple construction, the optional pairwise spacing constraint was disabled so that extremely close source combinations were not artificially excluded.

In the continuous-manifold joint refinement stage, the number of outer refinement rounds was set to Q=4. For each coordinate update, the refinement span was set to $\rho =0.6^{\circ }$, the initial parabolic step size was set to $h_{0}=0.10^{\circ }$, and the number of inner parabolic updates was fixed to ψ=3. The step size was then reduced geometrically using the shrinkage factor $\xi_{h}=0.5$, yielding a coarse-to-fine local search strategy with low computational overhead and stable convergence in close-angle scenarios.

To validate the performance of the proposed algorithm, we considered an 8-element array with 200 snapshots, and all subsequent experiments were conducted under the same array configuration unless otherwise stated. To evaluate the close-angle resolution capability, the first source was fixed at $10.55^{\circ }$, while the second source varied from $11.55^{\circ }$ to $20.55^{\circ }$ with an interval of $1^{\circ }$. For each source-spacing configuration under each SNR condition, the results were obtained from 100 Monte Carlo trials. The corresponding estimation results are summarized in Table 1.

For fairness, all compared methods were evaluated under the same signal model, array geometry, source-number setting, snapshot number, SNR range, and DOA configuration as the proposed method. For the deep-learning-based baselines, including the GNN- and CNN-based models, the training, validation, and test data were generated using the same simulation protocol as that used for the proposed method. The input data of these learning-based baselines were constructed from the same received array observations, and no additional oracle information, such as the true DOAs, ideal source amplitudes, or privileged noise-free covariance matrices, was provided during testing. The architectures and hyperparameters of the GNN and CNN baselines followed the settings reported in the corresponding reference, and they were kept fixed across the tested scenarios rather than being separately tuned for each individual test case. These settings were adopted to ensure that the performance differences mainly reflect the estimation and modeling capability of each method rather than inconsistent data generation or privileged prior information.

**Figure 1:**
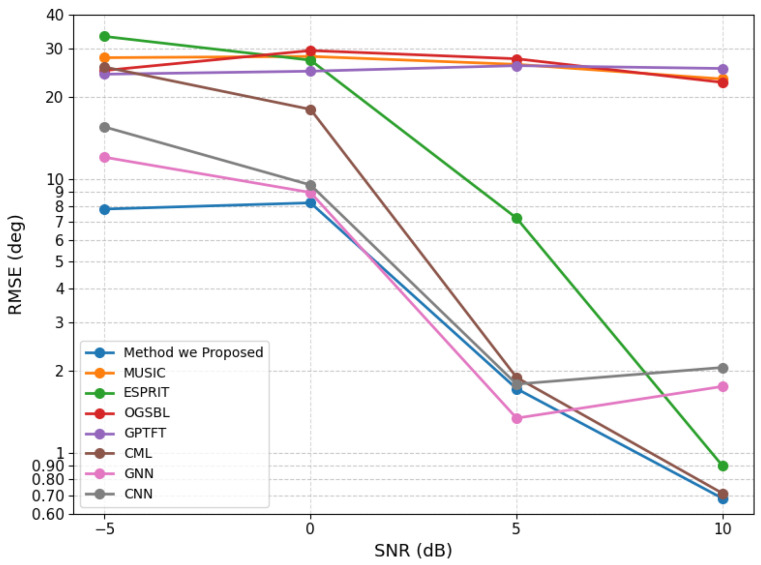
RMSE comparison of different methods under varying SNR conditions.

**Figure 2:**
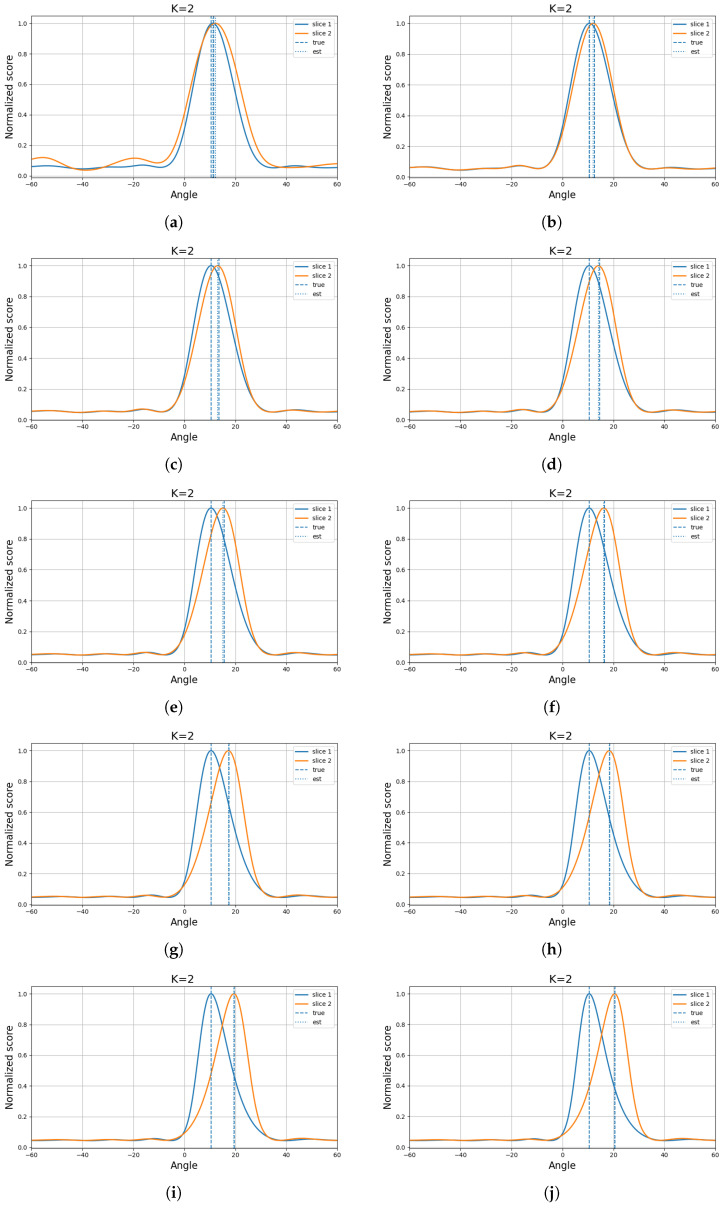
Spatial spectra of the proposed method at 10 dB for different source separations. The first source is fixed at $10.55^{\circ }$, while the second source varies from $11.55^{\circ }$ to $20.55^{\circ }$ with an interval of $1^{\circ }$. (**a**) $\left(10.55^{\circ },\;11.55^{\circ }\right)$. (**b**) $\left(10.55^{\circ },\;12.55^{\circ }\right)$. (**c**) $\left(10.55^{\circ },\;13.55^{\circ }\right)$. (**d**) $\left(10.55^{\circ },\;14.55^{\circ }\right)$. (**e**) $\left(10.55^{\circ },\;15.55^{\circ }\right)$. (**f**) $\left(10.55^{\circ },\;16.55^{\circ }\right)$. (**g**) $\left(10.55^{\circ },\;17.55^{\circ }\right)$. (**h**) $\left(10.55^{\circ },\;18.55^{\circ }\right)$. (**i**) $\left(10.55^{\circ },\;19.55^{\circ }\right)$. (**j**) $\left(10.55^{\circ },\;20.55^{\circ }\right)$.

For a comparative evaluation of different methods, the source angles were fixed at $\left(10.55^{\circ },\;12.55^{\circ }\right)$ under the setting of an eight-element array with 200 snapshots. For each method and each SNR level, the performance was evaluated over 100 Monte Carlo trials. As conventional baselines, three traditional algorithms, namely MUSIC, ESPRIT, and OGSBL, were considered. In addition, two deep-learning-based models with relatively strong performance under low-SNR and close-angle conditions were included [24,25]. To further assess the effectiveness of the proposed method in closely spaced scenarios, two representative algorithms specifically designed for close-angle DOA estimation, namely CML [26] and GPTFT [15], were also incorporated into the comparison. The corresponding experimental results are shown in Figure 1. In addition, the spectrum obtained at 10 dB is illustrated in Figure 2.

The experimental results show that the proposed method achieves the best overall performance among the compared algorithms. In particular, it outperforms all competing methods under all considered SNR conditions except at 5 dB, where the GNN-based model yields slightly lower RMSE. Nevertheless, the proposed method still performs better than the remaining algorithms at this SNR level, demonstrating its stability and superiority.

**Figure 3:**
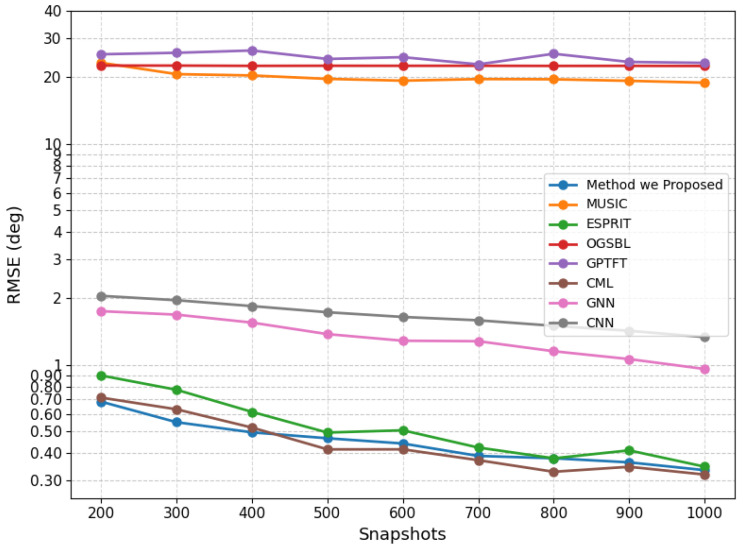
RMSE of the proposed method under different numbers of snapshots at 10 dB.

To further assess the effect of snapshot number on estimation performance, we conducted a comparative study involving multiple methods. The source angles were fixed at $\left(10.55^{\circ },\;12.55^{\circ }\right)$, and the SNR was set to 10 dB. For each snapshot setting ranging from 200 to 1000, the RMSE was computed from 100 Monte Carlo trials. The detailed results are summarized in Table 2 and Figure 3.

It can be observed that increasing the number of snapshots generally improves the estimation performance of all methods. The proposed method achieves superior performance over most competing approaches throughout the tested snapshot range. Specifically, it consistently yields lower RMSE than MUSIC, OGSBL, GPTFT, GNN, and CNN while also outperforming ESPRIT in most cases. Although CML becomes slightly more accurate under high-snapshot conditions, the proposed method remains highly competitive and demonstrates stable performance across all evaluated settings. These results further confirm the effectiveness of the proposed method in exploiting additional snapshots for close-angle DOA estimation.

**Figure 4:**
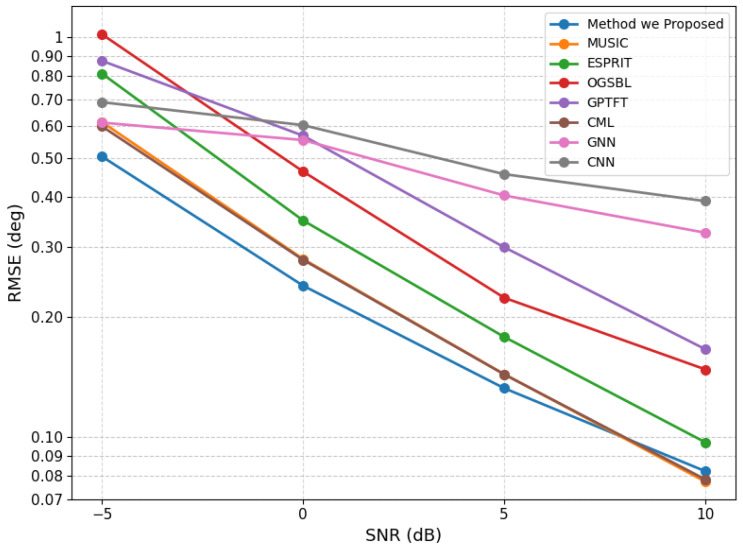
RMSE performance of the proposed method under a well-separated scenario at different SNR levels.

**Figure 5:**
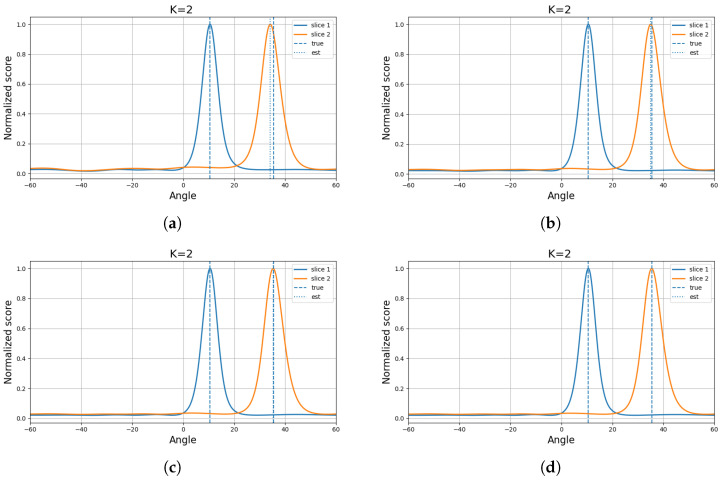
Spatial spectra of the proposed method under the well-separated scenario at different SNR levels. (**a**) SNR = −5 dB. (**b**) SNR = 0 dB. (**c**) SNR = 5 dB. (**d**) SNR = 10 dB.

We also evaluated the proposed model under a normal source-separation condition by fixing the source angles at ($10.55^{\circ }$, $35.54^{\circ }$) with 200 snapshots. For each SNR level, 100 Monte Carlo trials were performed, and the RMSE values were evaluated at −5, 0, 5, and 10 dB. The corresponding results are presented in Figure 4. The results show that except for a slight performance degradation relative to CML under the high-SNR condition, the proposed method outperforms all competing methods across the remaining SNR levels. Moreover, the spatial spectra under different SNR conditions are shown in Figure 5 to further illustrate the estimation behavior of the proposed method.

**Figure 6:**
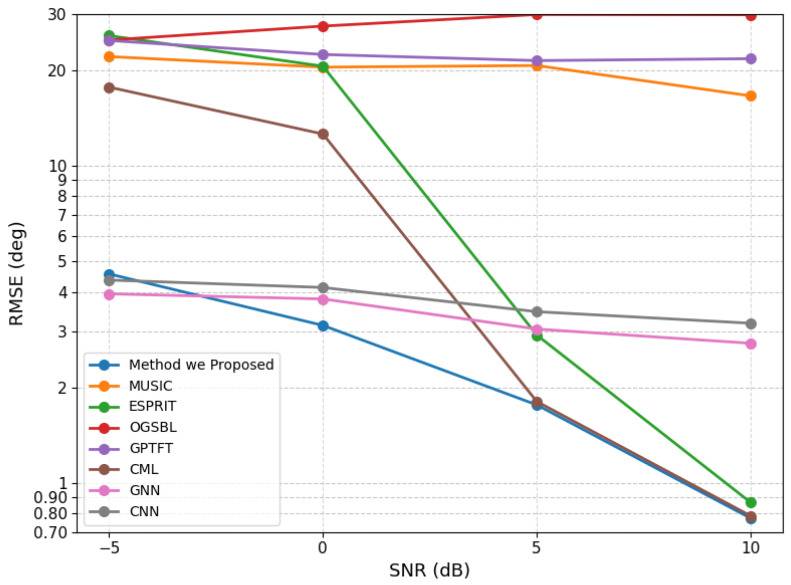
RMSE comparison of different methods versus SNR in the three-source scenario with three sources.

To further verify the effectiveness of the proposed method in a three-source scenario, additional experiments were conducted with the source angles fixed at $\left(-2.55^{\circ },\;5.55^{\circ },\;10.55^{\circ }\right)$. The number of snapshots was set to 200, and the SNR varied from −5 dB to 10 dB. For each SNR level, 100 Monte Carlo trials were performed, and the RMSE results were compared with those of several representative baseline methods. The corresponding quantitative results are summarized in Table 3, and the performance curves are shown in Figure 6.

It can be observed that the proposed method achieves competitive and generally superior RMSE performance under the tested SNR conditions. Although the GNN- and CNN-based baselines obtain slightly lower RMSE values at the lowest SNR point, the proposed method exhibits a clearer improvement trend as the SNR increases and achieves the best or near-best performance in the remaining cases. In particular, it maintains clear advantages over MUSIC, OGSBL, and GPTFT across the entire SNR range while also remaining highly competitive with ESPRIT, CML, GNN, and CNN in this three-source setting. More specifically, as the SNR increases from −5 dB to 10 dB, the RMSE of the proposed method decreases steadily from $4.55^{\circ }$ to $0.77^{\circ }$, indicating that the method can effectively exploit the improvement in noise conditions and preserve stable estimation behavior in the multi-source case. By contrast, several competing methods either remain at a relatively high error level over the whole SNR range or show noticeably weaker improvement trends, especially in the presence of three closely spaced sources. This phenomenon suggests that the proposed framework is better able to alleviate source interaction and reduce the risk of source merging when the estimation scene becomes more crowded and the coherence among steering vectors increases.

It is also worth noting that the proposed method shows a clear improvement trend as the SNR increases and achieves best or near-best performance over most tested SNR conditions. Such behavior reflects the benefit of combining MMV-based evidence learning, diversified candidate construction, and joint continuous refinement, which together provide improved robustness against both noise and multi-source interference. Therefore, these results demonstrate that the proposed method remains effective and robust even when the number of sources increases, confirming its capability for multi-source close-angle DOA estimation.

**Figure 7:**
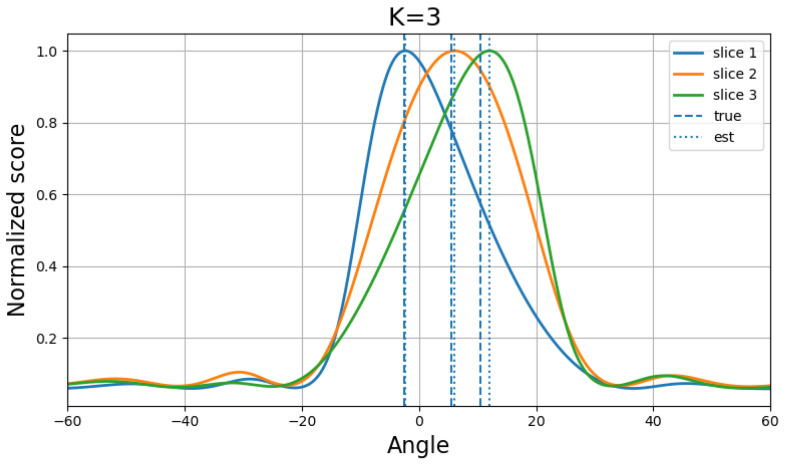
Spatial spectra at 10 dB in the three-source scenario with three sources.

The corresponding spatial spectra at 10 dB for this three-source case are shown in Figure 7, which further illustrates the superiority of the proposed method in resolving multiple closely spaced sources. In particular, the spectrum shows that the proposed framework is able to produce distinct responses around the three target directions rather than collapsing adjacent sources into an insufficient number of dominant peaks. This qualitative observation is consistent with the quantitative RMSE results and further supports the effectiveness of the proposed method in challenging three-source close-angle scenarios.

To further complement the RMSE-based evaluation, an additional accuracy (ACC) metric is introduced to measure the proportion of successful DOA estimation trials. In this metric, a trial is regarded as accurate only when the absolute errors of both estimated source angles are smaller than $1^{\circ }$ simultaneously. Therefore, ACC directly reflects whether the two closely spaced sources are successfully localized in each Monte Carlo trial rather than only measuring the average angular error. In this additional experiment, the two sources are fixed at $\left(-10.35^{\circ },-5.35^{\circ }\right)$, corresponding to a $5^{\circ }$ angular separation. The ACC results under different SNR levels are summarized in Table 4.

## 4. Discussion

The experimental results show that the proposed method is particularly effective in challenging close-angle and multi-source scenarios. Its main advantage is observed when adjacent steering vectors are highly coherent, where the Bayesian evidence learning, diversified candidate preservation, and joint refinement strategy can better retain neighboring source hypotheses and reduce the risk of source merging. However, the improvement is not uniform across all scenarios. In relatively favorable cases, such as well-separated sources, high-SNR conditions, or large-snapshot settings, classical or likelihood-based methods may also achieve competitive performance. This indicates that the proposed close-angle-oriented design is most beneficial when source separation is limited and the estimation landscape is strongly affected by coherence.

The snapshot-varying results further show that increasing the number of snapshots generally improves the estimation performance, since more snapshots provide more stable covariance and evidence information. Nevertheless, the advantage of the proposed method becomes less dominant when sufficient snapshots and favorable noise conditions are available. This observation suggests that the proposed framework should be interpreted as a robust close-angle enhancement strategy rather than a universally superior replacement for all DOA estimators. In very low-SNR or extremely small-separation cases, the evidence profile may still become unstable, and reliable resolution remains challenging.

A remaining limitation of the current framework is its relatively high computational complexity. Although the parabolic refinement is more efficient than dense grid search, the overall procedure still involves iterative MMV-SBL updates, candidate selection, multi-start initialization, and repeated joint refinement. More specifically, let *M* denote the number of sensors, *T* the number of snapshots, *G* the number of angular grid points, *I* the number of Bayesian evidence-learning iterations, $P_{\mathrm{cand}}$ the number of retained candidates, *K* the number of sources, *Q* the number of outer refinement rounds, and ψ the number of inner parabolic updates. The computational complexity of the Bayesian evidence-learning stage is approximately $O(MG^{2}+MGT+I\left(G^{3}+G^{2}T\right))$, while the candidate construction stage requires approximately $O(G\log G+GP_{\mathrm{cand}})$. The multi-start joint refinement stage evaluates the residual objective over candidate tuples, leading to a complexity of approximately OPcandKQψKMT. Therefore, the dominant computational cost can be summarized as OI(G3+G2T)+PcandKQψKMT. For the two-source case considered in most experiments, the refinement term reduces to approximately $O(P_{\mathrm{cand}}^{2}Q\psi MT)$.

To provide an illustrative runtime comparison, the mean runtime per trial was measured under the two-source close-angle setting. The OGSBL baseline requires approximately 2.2792 s per trial, while the proposed method requires approximately 6.7326 s per trial. The additional runtime mainly comes from the diversified candidate construction and multi-start joint continuous refinement after Bayesian evidence learning. This extra computational cost is the price paid for preserving multiple close-angle hypotheses and reducing the risk of source merging. Therefore, future work will focus on reducing the computational cost through adaptive candidate pruning, parallel multi-start refinement, and more efficient evidence-update strategies. Meanwhile, the method will also be extended to more realistic and diverse scenarios, such as model mismatch, colored noise, mutual coupling, sensor gain-phase errors, multi-path propagation, fewer-snapshot conditions, and larger-scale array configurations. In addition, measured-data validation will be considered to further evaluate the practical applicability of the proposed framework in real-world array processing environments.

Regarding generalization to more extreme scenarios, the proposed framework is expected to remain applicable when the angular separation becomes smaller or the number of snapshots is reduced, because its diversified candidate construction is designed to preserve multiple plausible hypotheses from highly coherent angular regions. However, its reliability will inevitably decrease when the separation is below the effective resolution capability of the array or when the snapshot number is too small to provide a stable covariance/evidence estimate. For example, when two sources are separated by less than $1^{\circ }$, the steering vectors become extremely coherent for an eight-element ULA, and the evidence profile may no longer contain sufficient information to reliably distinguish the two sources. Similarly, under very few snapshots, the MMV evidence learning stage may become less stable because the shared support information across snapshots is insufficient. Therefore, although the proposed method can alleviate source merging in challenging close-angle cases, extremely small angular separations and very limited snapshots remain difficult and should be regarded as important directions for future robustness enhancement.

## 5. Conclusions

In this paper, we proposed a Bayesian off-grid DOA estimation framework for close-angle and multi-source scenarios. By combining MMV-based evidence learning, diversified candidate selection, and multi-start joint refinement, the proposed method improves source discrimination and reduces source merging in high-coherence conditions. Simulation results under close-angle, well-separated, snapshot-varying, and three-source settings demonstrated its competitive and often superior estimation accuracy compared with representative baseline methods. A remaining limitation of the current framework is its relatively high computational complexity. Future work will therefore focus on reducing the computational cost while extending the method to more practical scenarios, such as model mismatch, colored noise, mutual coupling, sensor gain-phase errors, and larger-scale array configurations. In addition, measured-data validation will be considered to further evaluate the practical applicability of the proposed framework in real-world array processing environments.

## Figures and Tables

**Table 1:** RMSE of the proposed method under different angular separations and SNR.

True DOAs	10 dB	5 dB	0 dB	−5 dB
$\left(10.55^{\circ },\;11.55^{\circ }\right)$	2.1111	11.1330	14.5461	12.5716
$\left(10.55^{\circ },\;12.55^{\circ }\right)$	0.6821	1.7173	8.2094	7.7907
$\left(10.55^{\circ },\;13.55^{\circ }\right)$	0.4220	0.8939	2.2635	4.4854
$\left(10.55^{\circ },\;14.55^{\circ }\right)$	0.3135	0.6136	1.4493	3.4452
$\left(10.55^{\circ },\;15.55^{\circ }\right)$	0.2528	0.4789	1.0255	2.4276
$\left(10.55^{\circ },\;16.55^{\circ }\right)$	0.2121	0.3950	0.8078	1.9544
$\left(10.55^{\circ },\;17.55^{\circ }\right)$	0.1817	0.3352	0.6669	1.5597
$\left(10.55^{\circ },\;18.55^{\circ }\right)$	0.1577	0.2893	0.5653	1.2729
$\left(10.55^{\circ },\;19.55^{\circ }\right)$	0.1382	0.2526	0.4880	1.0751
$\left(10.55^{\circ },\;20.55^{\circ }\right)$	0.1223	0.2231	0.4276	0.9299

**Table 2:** RMSE comparison of different methods under different numbers of snapshots.

Snapshots	Proposed	MUSIC	ESPRIT	OGSBL	GPTFT	CML	GNN	CNN
200	0.6821	23.2838	0.8960	22.6180	25.4395	0.7115	1.7511	2.0541
300	0.5508	20.6839	0.7720	22.6117	25.8331	0.6300	1.6889	1.9627
400	0.4956	20.3991	0.6133	22.5465	26.4591	0.5208	1.5547	1.8463
500	0.4660	19.6847	0.4945	22.5715	24.2281	0.4151	1.3788	1.7318
600	0.4412	19.3210	0.5063	22.5607	24.6812	0.4152	1.2869	1.6489
700	0.3877	19.6457	0.4230	22.5651	22.9078	0.3709	1.2805	1.5924
800	0.3784	19.6009	0.3782	22.5359	25.5748	0.3287	1.1536	1.5037
900	0.3626	19.2899	0.4108	22.5458	23.4862	0.3462	1.0632	1.4276
1000	0.3343	18.9225	0.3467	22.5151	23.2547	0.3194	0.9589	1.3368

**Table 3:** RMSE comparison of different methods in the three-source scenario with source angles fixed at $\left(-2.55^{\circ },\;5.55^{\circ },\;10.55^{\circ }\right)$ and 200 snapshots.

SNR (dB)	Proposed	MUSIC	ESPRIT	OGSBL	GPTFT	CML	GNN	CNN
−5	4.55	22.05	25.67	24.87	24.77	17.66	3.94	4.36
0	3.13	20.42	20.56	27.50	22.38	12.57	3.80	4.13
5	1.76	20.66	2.92	29.92	21.41	1.80	3.05	3.46
10	0.77	16.58	0.87	29.87	21.71	0.79	2.75	3.18

**Table 4:** ACC of the proposed method under a representative close-angle scenario with source angles fixed at $\left(-10.35^{\circ },-5.35^{\circ }\right)$ and 200 snapshots.

Metric	SNR level			
SNR (dB)	10	5	0	−5
ACC (%)	100.00	95.00	50.00	22.00

## Data Availability

The data supporting the findings of this study are available from the corresponding author upon reasonable request.

## Abbreviations

The following abbreviations are used in this manuscript:

SNR	Signal-to-Noise Ratio
DOA	Direction of Arrival
RMSE	Root Mean Square Error
ULA	Uniform Linear Array
MMV	Multi-Measurement Vector
ARD	Automatic Relevance Determination
SBL	Sparse Bayesian Learning
SSE	Sum of Squared Errors
MUSIC	MUltiple SIgnal Classification
ESPRIT	Estimation of Signal Parameters via Rotational Invariance Techniques
OGSBL	Off-Grid Sparse Bayesian Learning
CML	Conditional Maximum Likelihood
GPTFT	Gradient Projection Test of Feasibility Technique
GNN	Graph Neural Network
CNN	Convolutional Neural Network
